# Poly[tetra­aqua-μ_3_-benzene-1,2-di­carboxyl­ato-μ_3_-bromido-penta-μ_2_-bromido-octa-μ_3_-isonicotinato-hepta­copper(I)trilanthanum(III)]

**DOI:** 10.1107/S1600536809014081

**Published:** 2009-04-22

**Authors:** Guo-Ming Wang, Zeng-Xin Li, Shu-Yun Xue, Hui-Luan Liu

**Affiliations:** a Department of Chemistry, Teachers’ College of Qingdao University, Shandong 266071, People’s Republic of China

## Abstract

A new lanthanum(III)–copper(I) heterometallic coordination polymer, [Cu_7_La_3_Br_6_(C_6_H_4_NO_2_)_8_(C_8_H_4_O_4_)(H_2_O)_4_]_*n*_, has been prepared by a hydro­thermal method. Of the three La atoms in the asymmetric unit, two are eight-coordinate with bicapped trigonal–prismatic configurations; the third is nine-coordinated and has a tricapped trigonal–prismatic coordination geometry. Of the seven Cu atoms, two are two-coordinate with CuBrN and CuN_2_ ligand sets, three have trigonal configurations, *viz.* CuBrN_2_, CuBr_2_N and CuBr_3_, while the remaining two adopt distorted tetra­hedral CuBr_3_N geometries. In the crystal structure, adjacent La centers are linked by isonicotinate (IN^−^) and benzene-1,2-dicarboxyl­ate ligands to form a two-dimensional La–carboxyl­ate layer in the *ab* plane. These layers are further inter­connected with each other by bridging [Cu(IN)_2_] motifs, leading to an unusual three-dimensional heterometallic Cu–halide–lanthanide–organic framework, with the inorganic [Cu_6_Br_6_]_*n*_ chains located in the resulting channels. Two Cu atoms are disordered over two positions, both with site occupancy factors of 0.80 and 0.20. O—H⋯O hydrogen bonding between water molecules and carboxylate O atoms helps to consolidate the crystal packing.

## Related literature

For background on the structures and applications of heterometallic lanthanide–transition metal (Ln–TM) coordination polymers, see: Benelli & Gatteschi (2002 ▶); Shibasaki & Yoshikawa (2002 ▶); Zhao, Cheng *et al.* (2004 ▶); Zhao, Chen *et al.* (2004 ▶); Guillou *et al.* (2006 ▶); Wang *et al.* (2006 ▶). For some examples of extended heterometallic Ln–TM architectures, see: Ren *et al.* (2003 ▶); Prasad *et al.* (2007 ▶); Cheng *et al.* (2008 ▶); Deng *et al.* (2008 ▶); Wang, Li *et al.* (2008 ▶). For the coordination modes of isonicotinate and benzene-1,2-dicarboxyl­ate ligands, see: Gu & Xue (2007 ▶); Wang, Duan *et al.* (2008 ▶).
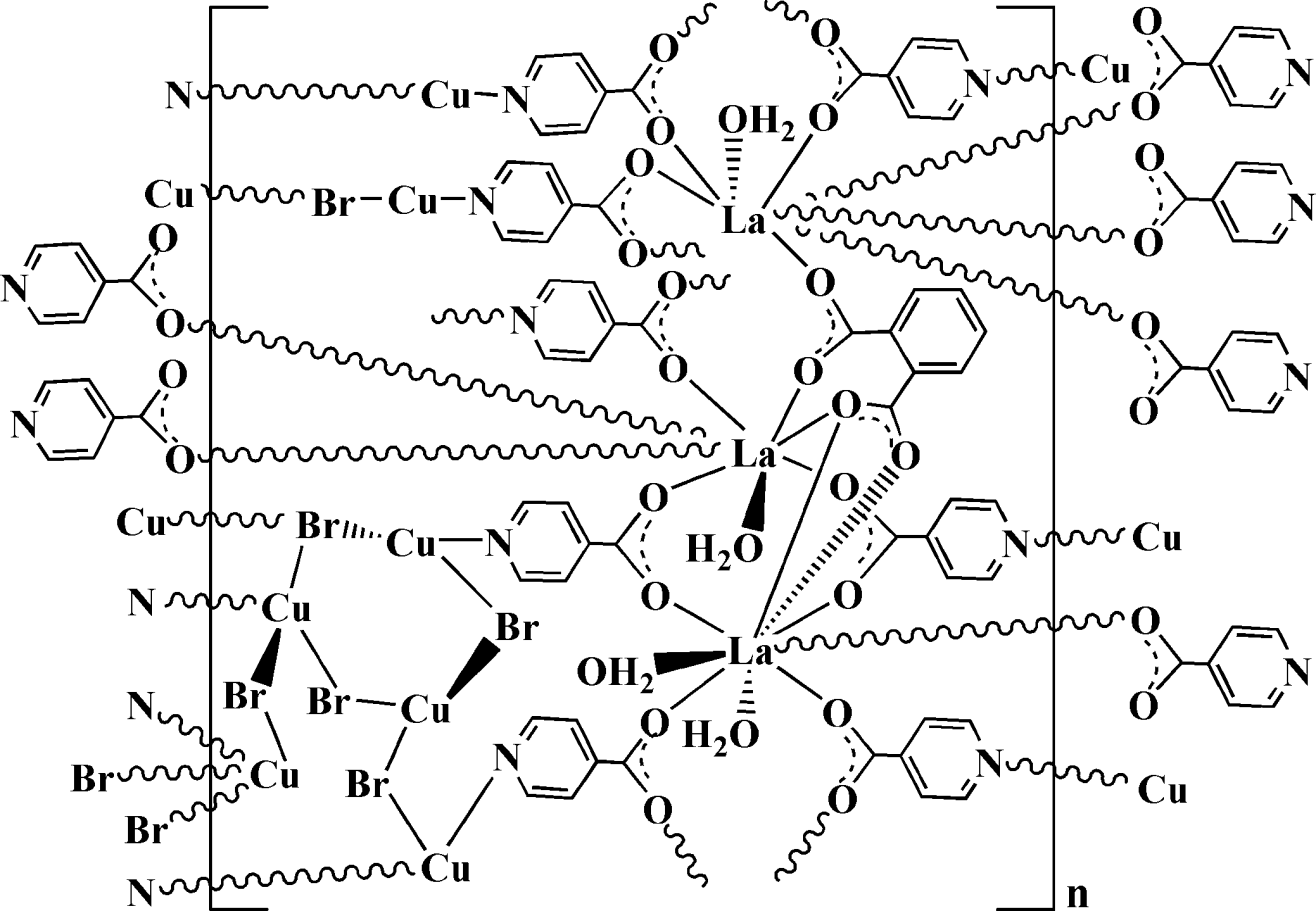


## Experimental

### 

#### Crystal data

[Cu_7_La_3_Br_6_(C_6_H_4_NO_2_)_8_(C_8_H_4_O_4_)(H_2_O)_4_]
*M* *_r_* = 2553.96
Monoclinic, 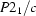

*a* = 10.1071 (5) Å
*b* = 19.6311 (3) Å
*c* = 34.4015 (2) Å
β = 92.480 (2)°
*V* = 6819.3 (4) Å^3^
*Z* = 4
Mo *K*α radiation
μ = 7.57 mm^−1^
*T* = 295 K
0.20 × 0.10 × 0.09 mm

#### Data collection

Bruker APEXII area-detector diffractometer
Absorption correction: multi-scan (*SADABS*; Sheldrick, 1996 ▶) *T*_min_ = 0.313, *T*_max_ = 0.549 (expected range = 0.288–0.506)
52743 measured reflections
13363 independent reflections
9870 reflections with *I* > 2σ(*I*)
*R*_int_ = 0.071

#### Refinement

*R*[*F* ^2^ > 2σ(*F* ^2^)] = 0.052
*wR*(*F* ^2^) = 0.126
*S* = 1.18
13363 reflections
955 parameters
H-atom parameters constrained
Δρ_max_ = 1.73 e Å^−3^
Δρ_min_ = −2.22 e Å^−3^

### 

Data collection: *APEX2* (Bruker, 2002 ▶); cell refinement: *SAINT* (Bruker, 2002 ▶); data reduction: *SAINT*; program(s) used to solve structure: *SHELXS97* (Sheldrick, 2008 ▶); program(s) used to refine structure: *SHELXL97* (Sheldrick, 2008 ▶); molecular graphics: *SHELXTL* (Sheldrick, 2008 ▶); software used to prepare material for publication: *SHELXL97*.

## Supplementary Material

Crystal structure: contains datablocks global, I. DOI: 10.1107/S1600536809014081/sj2614sup1.cif

Structure factors: contains datablocks I. DOI: 10.1107/S1600536809014081/sj2614Isup2.hkl

Additional supplementary materials:  crystallographic information; 3D view; checkCIF report

## Figures and Tables

**Table 1:** Hydrogen-bond geometry (Å, °)

*D*—H⋯*A*	*D*—H	H⋯*A*	*D*⋯*A*	*D*—H⋯*A*
O16—H16*D*⋯O21^i^	0.86	2.09	2.901 (7)	157
O23—H23*D*⋯O22	0.93	2.00	2.861 (8)	153
O24—H24*D*⋯O20	0.85	2.22	2.844 (11)	130

Symmetry code: (i) 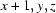.

**Fig. 1.**
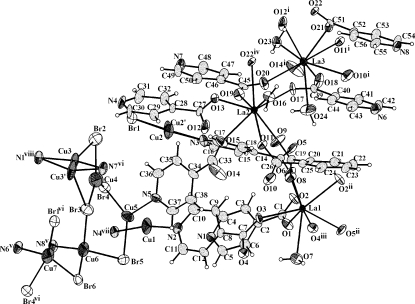
The molecular structure of 1, showing the atom-numbering scheme. Displacement ellipsoids are drawn at the 50% probability level. [Symmetry codes: (i) -1 + x, y, z; (ii) -x, 1 - y, - z; (iii) 1 - x, 1 - y, - z; (iv) -x, - y, - z; (v) 1 + x, 1/2 - y, -1/2 + z; (vi) 1 + x, y, z; (vii) 1 - x, 1/2 + y, -1/2 - z; (viii) 1 - x, -1/2 + y, -1/2 - z.]

**Fig. 2.**
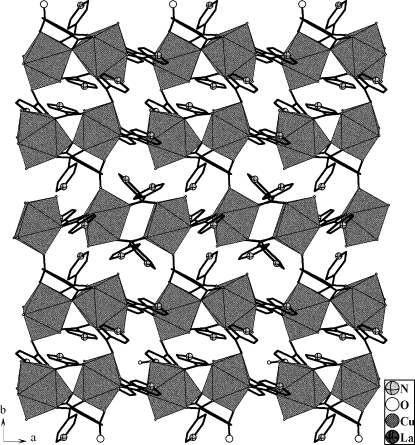
Two-dimensional La-carboxylate layer in the ab plane.

**Fig. 3.**
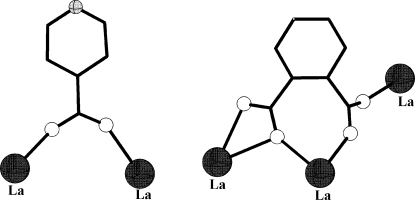
The coordination modes of IN- and BDC2- found in the La-carboxylate layer.

**Fig. 4.**
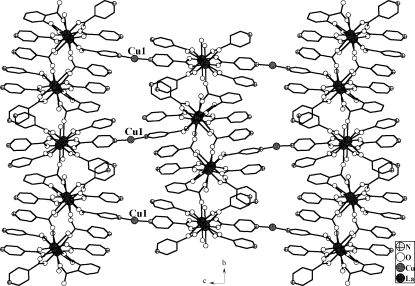
A view of the three-dimensional Cu-halide-lanthanide-organic framework with one-dimensional channels.

**Fig. 5.**
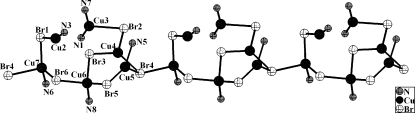
One-dimensional infinite [Cu6Br6]n chains along the a axis.

**Fig. 6.**
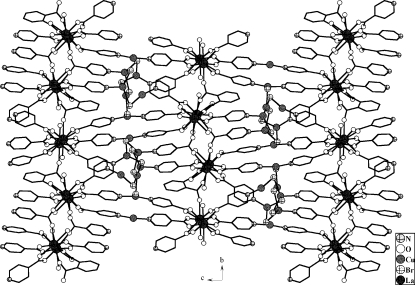
Framework of 1 viewed along the a axis.

## supplementary crystallographic information

### Comment

In recent years, the design and constructinon of heterometallic lanthanide(Ln)-transition metal(TM) coordination frameworks have attracted considerable attention because of their intriguing topological architectures and potential applications in for example magnetism, luminescence, and heterogeneous catalysis (Benelli & Gatteschi, 2002; Shibasaki & Yoshikawa, 2002; Zhao, Cheng *et al.*, 2004; Zhao, Chen *et al.*, 2004; Guillou *et al.*, 2006; Wang *et al.*, 2006). Compared with the assembly of homometallic Ln and TM compounds, the analogous chemistry and synthetic stategy of heterometallic Ln—TM coordination frameworks is still underdeveloped. (Ren *et al.*, 2003; Prasad *et al.*, 2007; Cheng *et al.*, 2008; Deng *et al.* (2008); Wang, Li *et al.* (2008). This may be attributed to the variable and versatile coordination numbers of the lanthanide ions, their low stereochemical preference, as well as the competitive reactions of Ln and TM metals for the same organic ligands. Fortunately, according to the hard-soft acid base theory, the Ln and TM ions have different affinities for O and N donors, which makes it possible to construct unusual heterometallic Ln—TM frameworks by choosing multifunctional ligands with both oxygen and nitrogen donors. Therefore, isonicotinic acid (HIN) has been chosen here as the bifunctional ligand.  Meanwhile, we also introduced another multifunctional ligand, the deprotonated 1,2-benzenedicarboxylic acid, (BDC^2-^), into the reaction system simultaneously, exploring the construction of heterometallic Ln—TM compounds with high dimensionality. The title compound [La_3_Cu_7_Br_6_(IN)_8_(BDC)(H_2_O)_4_]_n_ (1) is reported here and displays novel three-dimensional coordination features.

As shown in Fig. 1, the asymmetric unit contains three unique lanthanum(III) atoms, seven copper(I) ions, six bromide ions, one BDC^2-^ ligand and eight IN^-^ ligands, as well as four aqua ligands. The La1 and La2 atoms are both eight-coordinate with bicapped trigonal prismatic geometries. Atom La1 is surrounded by six carboxylate oxygen atoms from six IN^-^ ligands, one carboxylate oxygen atom from a BDC^2-^ ligand and an aqua ligand. Atom La2, on the other hand, is coordinated by five carboxylate oxygen atoms from five IN^-^ ligands, two carboxylate oxygen atoms from a BDC^2-^ ligand and an aqua ligand. The La3 atom is nine-coordinated and has a tricapped trigonal-prismatic coordination environment comprising two coordinated water molecules, five carboxylate oxygen atoms from five IN^-^ ligands and two carboxylate oxygen atoms from one BDC^2-^ ligand. The La—O bond lengths range from 2.387 (6) to 2.797 (6)Å.

The identical La(III) ions are linked by mixed IN^-^ and BDC^2-^ ligands to form a two-dimensional La-carboxylate layer in the *ab* plane (Fig. 2). Interestingly, compared to the abundant and versatile coordination modes that found in IN^-^ and BDC^2-^ ligands (Gu & Xue, 2007; Wang, Duan *et al.*, 2008), only a single bidentate (for IN^-^) and a unique pentadentate (for BDC^2-^) modes are adopted in the La-carboxylate layer (Fig. 3). These La-carboxylate layers are further interconnected by [Cu(1)(IN)_2_] linear bridging to give rise to an unusual Cu-halide-lanthanide-organic framework with one-dimensional channels (Fig. 4), in which the inorganic [Cu_6_Br_6_]_n_ chains are located. As shown in Fig. 5, the inorganic [Cu_6_Br_6_]_n_ motif contains six unique Cu(I)atoms with three different types of coordination modes and six Br^-^ ions. The Cu2 atom is two-coordinated with a neraly linear geometry: one µ_2_-Br1 ion and one N atom from one bridging IN^-^ ligand. The Cu3, Cu4 and Cu5 atoms are three-coordinate with trigonal coordination environments: two isonicotinate N atoms and one µ_2_-Br2 ion are bonded to Cu3; one µ_2_-Br2 ion, one µ_3_-Br3 ion and one µ_3_-Br4 ion to Cu4; one µ_2_-Br5 ion, one µ_3_-Br4 ion and one N atom to Cu5. The remaining Cu6 and Cu7 atoms, however, are coordinated to one N atom and three µ_2_-Br (Br3, Br5 and Br6 for Cu6; Br1, Br4 and Br6 for Cu7) ions respectively, defining distorted tetrahedral geometries. The Cu—N and Cu—Br distances are in the range 1.921 (7)–2.031 (8) Å and 2.228 (2)–2.670 (2) Å, respectively. Therefore, the overall structure of 1 can also be viewed as one-dimensional [Cu_6_Br_6_]_n_ chains inserted into the channels of a three-dimensional heterometallic Cu-halide-lanthanide-organic framework (Fig. 6).

### Experimental

The title compound was synthesized under mild hydrothermal conditions. Typically, a mixture of La_2_O_3_ (0.5 mmol; 0.163 g), CuBr_2_ (0.067 g, 0.30 mmol), HIN (2.00 mmol, 0247 g), H_2_BDC (1.00 mmol, 0.167 g) and H_2_O (8 ml) was sealed in a 25 ml Teflon-lined steel autoclave and heated under autogenous pressure at 443 K for 9 days. The brown prism-like crystals obtained were recovered by filtration, washed with distilled water and dried in air. Although copper(II) salts were used as starting materials, the Cu centers in the product are in the +1 oxidation state. This is attributed to a reduction reaction occurring under the hydrothermal conditions used.

### Refinement

H atoms bound to C atoms were positioned geometrically, with C—H distances of 0.93 Å, and constrained to ride on their parent atoms [*U*_iso_(H) = 1.2*U*_eq_(C)]. H atoms bound to O atoms were located in a difference Fourier map and treated as riding, with *U*_iso_(H) = 1.2*U*_eq_(O). Atoms Cu2 and Cu3 were refined as disordered over two positions, with site occupancy factors of fixed at 0.80 and 0.20 respectively in the final refinement.

### Crystal data

**Table:**

[Cu_7_La_3_Br_6_(C_6_H_4_NO_2_)_8_(C_8_H_4_O_4_)(H_2_O)_4_]	*F*(000) = 4848
*M**_r_* = 2553.96	*D*_x_ = 2.488 Mg m^−^^3^
Monoclinic, *P*2_1_/*c*	Mo *K*α radiation, λ = 0.71073 Å
Hall symbol: -P 2ybc	Cell parameters from 52743 reflections
*a* = 10.1071 (5) Å	θ = 1.2–26.0°
*b* = 19.6311 (3) Å	µ = 7.57 mm^−^^1^
*c* = 34.4015 (2) Å	*T* = 295 K
β = 92.480 (2)°	Prism, brown
*V* = 6819.3 (4) Å^3^	0.20 × 0.10 × 0.09 mm
*Z* = 4	

### Data collection

**Table:**

Bruker APEXII area-detector diffractometer	13363 independent reflections
Radiation source: fine-focus sealed tube	9870 reflections with *I* > 2σ(*I*)
graphite	*R*_int_ = 0.071
φ and ω scans	θ_max_ = 26.0°, θ_min_ = 1.2°
Absorption correction: multi-scan (*SADABS*; Sheldrick, 1996)	*h* = −12→12
*T*_min_ = 0.313, *T*_max_ = 0.549	*k* = −23→24
52743 measured reflections	*l* = −42→42

### Refinement

**Table:**

Refinement on *F*^2^	Primary atom site location: structure-invariant direct methods
Least-squares matrix: full	Secondary atom site location: difference Fourier map
*R*[*F*^2^ > 2σ(*F*^2^)] = 0.052	Hydrogen site location: inferred from neighbouring sites
*wR*(*F*^2^) = 0.126	H-atom parameters constrained
*S* = 1.18	*w* = 1/[σ^2^(*F*_o_^2^) + (0.0498*P*)^2^ + 4.7192*P*] where *P* = (*F*_o_^2^ + 2*F*_c_^2^)/3
13363 reflections	(Δ/σ)_max_ = 0.001
955 parameters	Δρ_max_ = 1.73 e Å^−^^3^
0 restraints	Δρ_min_ = −2.22 e Å^−^^3^

### Special details

**Table:**

Geometry. All esds (except the esd in the dihedral angle between two l.s. planes) are estimated using the full covariance matrix. The cell esds are taken into account individually in the estimation of esds in distances, angles and torsion angles; correlations between esds in cell parameters are only used when they are defined by crystal symmetry. An approximate (isotropic) treatment of cell esds is used for estimating esds involving l.s. planes.
Refinement. Refinement of F^2^ against ALL reflections. The weighted R-factor wR and goodness of fit S are based on F^2^, conventional R-factors R are based on F, with F set to zero for negative F^2^. The threshold expression of F^2^ > 2sigma(F^2^) is used only for calculating R-factors(gt) etc. and is not relevant to the choice of reflections for refinement. R-factors based on F^2^ are statistically about twice as large as those based on F, and R- factors based on ALL data will be even larger.

### Fractional atomic coordinates and isotropic or equivalent isotropic displacement parameters (Å^2^)

**Table:**

	*x*	*y*	*z*	*U*_iso_*/*U*_eq_	Occ. (<1)
La1	0.22600 (4)	0.48720 (2)	0.006803 (12)	0.01347 (11)	
La2	0.29679 (4)	0.14367 (2)	−0.026957 (12)	0.01318 (11)	
La3	−0.24624 (4)	0.16813 (2)	−0.017184 (13)	0.01663 (12)	
Cu1	0.38381 (16)	0.52109 (8)	−0.24321 (3)	0.0605 (4)	
Cu2	0.1352 (4)	0.2896 (2)	−0.21115 (11)	0.0565 (9)	0.80
Cu2'	0.0878 (17)	0.3052 (9)	−0.2218 (4)	0.074 (5)	0.20
Cu3	0.8350 (3)	0.1565 (2)	−0.26477 (12)	0.0440 (7)	0.80
Cu3'	0.8908 (14)	0.1708 (11)	−0.2644 (5)	0.064 (4)	0.20
Cu4	0.63663 (16)	0.28675 (8)	−0.26968 (5)	0.0653 (4)	
Cu5	0.61139 (14)	0.41101 (7)	−0.23317 (4)	0.0530 (4)	
Cu6	0.88795 (15)	0.43063 (7)	−0.27424 (3)	0.0514 (4)	
Cu7	1.21736 (16)	0.34075 (8)	−0.28116 (4)	0.0576 (4)	
Br1	0.21910 (10)	0.22413 (5)	−0.25622 (3)	0.0408 (3)	
Br2	0.57870 (12)	0.17237 (6)	−0.26488 (4)	0.0548 (3)	
Br3	0.85917 (10)	0.30796 (5)	−0.25766 (3)	0.0443 (3)	
Br4	0.47465 (9)	0.36921 (5)	−0.28606 (3)	0.0359 (2)	
Br5	0.76335 (9)	0.50127 (5)	−0.23036 (3)	0.0380 (2)	
Br6	1.13084 (9)	0.44473 (5)	−0.25274 (3)	0.0362 (2)	
O1	0.1380 (6)	0.5739 (3)	−0.04055 (17)	0.0325 (15)	
O2	−0.0776 (5)	0.5648 (3)	−0.05687 (16)	0.0269 (14)	
O3	0.3829 (5)	0.4716 (3)	−0.04459 (15)	0.0279 (14)	
O4	0.5802 (6)	0.5205 (3)	−0.05111 (16)	0.0308 (15)	
O5	−0.1574 (6)	0.4261 (3)	−0.05461 (18)	0.0383 (17)	
O6	0.0482 (6)	0.4339 (3)	−0.03153 (17)	0.0392 (17)	
O7	0.3772 (6)	0.6005 (3)	0.00383 (19)	0.0406 (17)	
H7A	0.4177	0.5917	−0.0167	0.080*	
H7B	0.4321	0.5951	0.0231	0.080*	
O8	0.2809 (6)	0.3656 (3)	0.00780 (17)	0.0325 (15)	
O9	0.2517 (7)	0.2573 (3)	−0.00553 (19)	0.0432 (18)	
O10	0.6667 (6)	0.2366 (4)	0.0380 (2)	0.050 (2)	
O11	0.4973 (6)	0.1853 (3)	0.00866 (16)	0.0275 (14)	
O12	0.6320 (6)	0.0893 (4)	−0.06027 (17)	0.0433 (18)	
O13	0.4174 (5)	0.0698 (3)	−0.07032 (17)	0.0306 (15)	
O14	0.6171 (7)	0.2426 (4)	−0.0589 (2)	0.064 (3)	
O15	0.4104 (6)	0.2149 (3)	−0.07631 (18)	0.0381 (16)	
O16	0.4042 (5)	0.0575 (3)	0.02259 (16)	0.0295 (14)	
H16C	0.3978	0.0251	0.0388	0.080*	
H16D	0.4832	0.0734	0.0256	0.080*	
O17	0.1231 (5)	0.1276 (3)	0.01773 (16)	0.0285 (14)	
O18	−0.0733 (6)	0.1657 (4)	0.03231 (17)	0.0381 (17)	
O19	0.1224 (6)	0.1580 (3)	−0.07778 (18)	0.0331 (15)	
O20	−0.0813 (6)	0.1887 (4)	−0.06543 (17)	0.0381 (16)	
O21	−0.3099 (5)	0.0712 (3)	0.02882 (15)	0.0217 (13)	
O22	−0.1878 (5)	−0.0234 (3)	0.03169 (15)	0.0197 (12)	
O23	−0.1039 (5)	0.0580 (3)	−0.03168 (17)	0.0321 (15)	
H23C	−0.0254	0.0630	−0.0290	0.080*	
H23D	−0.1202	0.0204	−0.0159	0.080*	
O24	−0.1433 (10)	0.2896 (4)	−0.0098 (3)	0.084 (3)	
H24C	−0.1207	0.3284	−0.0007	0.101*	
H24D	−0.0999	0.2827	−0.0300	0.101*	
C1	0.0388 (8)	0.5791 (4)	−0.0635 (2)	0.0238 (19)	
C2	0.0667 (7)	0.6024 (4)	−0.1045 (2)	0.0205 (18)	
C3	−0.0229 (8)	0.5899 (5)	−0.1351 (2)	0.030 (2)	
H3A	−0.1032	0.5689	−0.1305	0.036*	
C4	0.0066 (9)	0.6087 (5)	−0.1727 (3)	0.037 (2)	
H4A	−0.0560	0.6005	−0.1928	0.044*	
C5	0.2079 (10)	0.6501 (5)	−0.1517 (3)	0.040 (3)	
H5A	0.2882	0.6708	−0.1567	0.049*	
C6	0.1829 (9)	0.6333 (5)	−0.1140 (3)	0.034 (2)	
H6A	0.2463	0.6431	−0.0944	0.040*	
C7	0.4703 (8)	0.5001 (4)	−0.0643 (2)	0.0206 (18)	
C8	0.4435 (8)	0.5066 (4)	−0.1068 (2)	0.0238 (19)	
C9	0.3389 (9)	0.4730 (5)	−0.1256 (2)	0.032 (2)	
H9A	0.2799	0.4476	−0.1115	0.038*	
C10	0.3229 (10)	0.4776 (5)	−0.1654 (3)	0.044 (3)	
H10A	0.2540	0.4534	−0.1777	0.052*	
C11	0.5003 (10)	0.5473 (5)	−0.1686 (3)	0.041 (3)	
H11A	0.5567	0.5733	−0.1833	0.050*	
C12	0.5248 (9)	0.5449 (5)	−0.1292 (2)	0.030 (2)	
H12A	0.5955	0.5688	−0.1177	0.035*	
C13	−0.0358 (8)	0.4176 (4)	−0.0570 (2)	0.0199 (18)	
C14	0.0095 (8)	0.3870 (4)	−0.0943 (2)	0.0205 (18)	
C15	−0.0494 (9)	0.4072 (5)	−0.1290 (3)	0.032 (2)	
H15A	−0.1170	0.4394	−0.1295	0.038*	
C16	−0.0078 (10)	0.3795 (5)	−0.1629 (3)	0.039 (2)	
H16A	−0.0463	0.3949	−0.1864	0.047*	
C17	0.1388 (9)	0.3108 (5)	−0.1299 (3)	0.039 (3)	
H17A	0.2010	0.2759	−0.1301	0.047*	
C18	0.1073 (8)	0.3381 (4)	−0.0946 (3)	0.030 (2)	
H18A	0.1508	0.3240	−0.0716	0.036*	
C19	0.2871 (8)	0.3047 (4)	0.0165 (2)	0.0218 (18)	
C20	0.3417 (8)	0.2869 (4)	0.0570 (2)	0.0220 (19)	
C21	0.2682 (8)	0.3108 (4)	0.0879 (3)	0.027 (2)	
H21A	0.1893	0.3341	0.0827	0.032*	
C22	0.3115 (9)	0.3002 (4)	0.1262 (3)	0.032 (2)	
H22A	0.2605	0.3151	0.1464	0.038*	
C23	0.4283 (9)	0.2680 (5)	0.1342 (3)	0.034 (2)	
H23A	0.4574	0.2609	0.1599	0.040*	
C24	0.5047 (9)	0.2454 (5)	0.1040 (3)	0.033 (2)	
H24A	0.5864	0.2251	0.1098	0.039*	
C25	0.4602 (7)	0.2528 (4)	0.0652 (2)	0.0211 (18)	
C26	0.5453 (8)	0.2248 (4)	0.0353 (3)	0.0244 (19)	
C27	0.5335 (8)	0.0750 (4)	−0.0816 (2)	0.0197 (18)	
C28	0.5547 (8)	0.0625 (4)	−0.1237 (2)	0.0201 (18)	
C29	0.6764 (8)	0.0441 (5)	−0.1370 (3)	0.032 (2)	
H29A	0.7490	0.0395	−0.1196	0.038*	
C30	0.6903 (9)	0.0325 (5)	−0.1760 (2)	0.033 (2)	
H30A	0.7731	0.0192	−0.1841	0.039*	
C31	0.4752 (10)	0.0582 (6)	−0.1901 (3)	0.045 (3)	
H31A	0.4060	0.0645	−0.2085	0.054*	
C32	0.4512 (9)	0.0690 (5)	−0.1518 (3)	0.038 (2)	
H32A	0.3666	0.0805	−0.1444	0.045*	
C33	0.5163 (9)	0.2446 (5)	−0.0812 (3)	0.031 (2)	
C34	0.5301 (8)	0.2866 (4)	−0.1169 (2)	0.0242 (19)	
C35	0.4653 (9)	0.2684 (5)	−0.1515 (3)	0.032 (2)	
H35A	0.4074	0.2315	−0.1525	0.038*	
C36	0.4877 (9)	0.3059 (5)	−0.1848 (3)	0.037 (2)	
H36A	0.4469	0.2921	−0.2083	0.045*	
C37	0.6226 (9)	0.3787 (5)	−0.1509 (3)	0.033 (2)	
H37A	0.6742	0.4179	−0.1500	0.040*	
C38	0.6105 (9)	0.3424 (4)	−0.1166 (3)	0.031 (2)	
H38A	0.6563	0.3559	−0.0939	0.038*	
C39	0.0442 (8)	0.1505 (4)	0.0412 (2)	0.0208 (18)	
C40	0.0887 (7)	0.1565 (4)	0.0830 (2)	0.0195 (18)	
C41	0.0109 (8)	0.1899 (4)	0.1090 (2)	0.027 (2)	
H41A	−0.0680	0.2106	0.1006	0.032*	
C42	0.0531 (9)	0.1919 (5)	0.1480 (3)	0.035 (2)	
H42A	0.0019	0.2150	0.1656	0.042*	
C43	0.2389 (9)	0.1316 (5)	0.1347 (3)	0.033 (2)	
H43A	0.3188	0.1123	0.1434	0.040*	
C44	0.2062 (8)	0.1270 (5)	0.0962 (3)	0.030 (2)	
H44A	0.2609	0.1048	0.0792	0.036*	
C45	0.0064 (8)	0.1715 (4)	−0.0876 (2)	0.0199 (18)	
C46	−0.0323 (8)	0.1683 (4)	−0.1304 (2)	0.0214 (18)	
C47	−0.1537 (8)	0.1923 (4)	−0.1445 (2)	0.027 (2)	
H47A	−0.2133	0.2114	−0.1277	0.033*	
C48	−0.1848 (9)	0.1875 (5)	−0.1837 (3)	0.033 (2)	
H48A	−0.2639	0.2070	−0.1930	0.040*	
C49	0.0058 (9)	0.1337 (5)	−0.1953 (2)	0.032 (2)	
H49A	0.0608	0.1126	−0.2126	0.038*	
C50	0.0497 (8)	0.1386 (4)	−0.1569 (3)	0.027 (2)	
H50A	0.1330	0.1224	−0.1489	0.032*	
C51	−0.2364 (7)	0.0287 (4)	0.0465 (2)	0.0179 (17)	
C52	−0.2047 (7)	0.0423 (4)	0.0886 (2)	0.0179 (17)	
C53	−0.2863 (8)	0.0798 (5)	0.1118 (3)	0.033 (2)	
H53A	−0.3620	0.1001	0.1007	0.039*	
C54	−0.2575 (10)	0.0875 (5)	0.1509 (3)	0.040 (3)	
H54A	−0.3163	0.1113	0.1659	0.048*	
C55	−0.0661 (9)	0.0266 (5)	0.1462 (3)	0.035 (2)	
H55A	0.0116	0.0091	0.1577	0.042*	
C56	−0.0915 (8)	0.0148 (5)	0.1070 (2)	0.029 (2)	
H56A	−0.0334	−0.0114	0.0930	0.035*	
N1	0.1216 (7)	0.6380 (4)	−0.1813 (2)	0.0334 (19)	
N2	0.4013 (8)	0.5150 (4)	−0.1872 (2)	0.040 (2)	
N3	0.0842 (9)	0.3320 (4)	−0.1638 (2)	0.043 (2)	
N4	0.5940 (8)	0.0390 (4)	−0.2026 (2)	0.0356 (19)	
N5	0.5652 (7)	0.3610 (4)	−0.1848 (2)	0.0315 (18)	
N6	0.1655 (8)	0.1616 (4)	0.1610 (2)	0.0346 (19)	
N7	−0.1092 (8)	0.1570 (4)	−0.2089 (2)	0.0346 (19)	
N8	−0.1468 (8)	0.0616 (4)	0.1681 (2)	0.0337 (19)	

### Atomic displacement parameters (Å^2^)

**Table:**

	*U*^11^	*U*^22^	*U*^33^	*U*^12^	*U*^13^	*U*^23^
La1	0.0140 (2)	0.0170 (2)	0.0094 (2)	0.00099 (17)	0.00035 (17)	0.00074 (18)
La2	0.0137 (2)	0.0148 (2)	0.0109 (2)	0.00006 (18)	−0.00093 (17)	−0.00007 (18)
La3	0.0145 (2)	0.0225 (3)	0.0126 (2)	0.00030 (19)	−0.00268 (17)	0.00349 (19)
Cu1	0.0995 (12)	0.0690 (11)	0.0132 (6)	0.0036 (9)	0.0041 (7)	0.0086 (6)
Cu2	0.075 (2)	0.055 (2)	0.041 (2)	−0.0055 (15)	0.0266 (14)	−0.0164 (14)
Cu2'	0.114 (13)	0.076 (9)	0.037 (8)	0.019 (8)	0.046 (8)	−0.005 (6)
Cu3	0.058 (2)	0.0613 (18)	0.0126 (10)	0.0103 (15)	−0.0006 (14)	−0.0047 (10)
Cu3'	0.093 (12)	0.086 (12)	0.014 (4)	0.031 (9)	−0.001 (8)	−0.006 (6)
Cu4	0.0700 (10)	0.0659 (11)	0.0600 (10)	−0.0001 (8)	0.0015 (8)	0.0028 (8)
Cu5	0.0697 (9)	0.0580 (9)	0.0309 (7)	−0.0293 (8)	−0.0008 (6)	0.0128 (6)
Cu6	0.0762 (10)	0.0571 (9)	0.0201 (7)	0.0036 (7)	−0.0047 (6)	0.0050 (6)
Cu7	0.0823 (11)	0.0660 (10)	0.0236 (7)	−0.0031 (8)	−0.0095 (7)	0.0041 (6)
Br1	0.0451 (6)	0.0387 (6)	0.0395 (6)	−0.0013 (5)	0.0128 (5)	−0.0057 (5)
Br2	0.0543 (7)	0.0558 (8)	0.0539 (7)	−0.0024 (6)	−0.0033 (6)	−0.0041 (6)
Br3	0.0484 (6)	0.0371 (6)	0.0475 (6)	0.0005 (5)	0.0032 (5)	0.0082 (5)
Br4	0.0291 (5)	0.0463 (6)	0.0317 (5)	−0.0051 (4)	−0.0062 (4)	0.0062 (5)
Br5	0.0354 (5)	0.0380 (6)	0.0405 (6)	−0.0063 (4)	0.0006 (4)	−0.0055 (5)
Br6	0.0335 (5)	0.0523 (7)	0.0230 (5)	−0.0105 (4)	0.0018 (4)	−0.0016 (4)
O1	0.029 (3)	0.042 (4)	0.026 (3)	−0.003 (3)	−0.006 (3)	0.020 (3)
O2	0.016 (3)	0.034 (4)	0.030 (3)	0.003 (3)	0.007 (3)	0.016 (3)
O3	0.028 (3)	0.042 (4)	0.014 (3)	−0.007 (3)	0.010 (2)	0.000 (3)
O4	0.028 (3)	0.041 (4)	0.022 (3)	−0.009 (3)	−0.009 (3)	0.003 (3)
O5	0.039 (4)	0.037 (4)	0.039 (4)	−0.002 (3)	0.005 (3)	−0.025 (3)
O6	0.054 (4)	0.039 (4)	0.024 (4)	−0.004 (3)	−0.014 (3)	−0.010 (3)
O7	0.032 (4)	0.044 (4)	0.045 (4)	−0.001 (3)	0.002 (3)	0.003 (3)
O8	0.048 (4)	0.020 (4)	0.028 (4)	0.001 (3)	−0.006 (3)	0.003 (3)
O9	0.063 (5)	0.017 (4)	0.047 (4)	0.015 (3)	−0.025 (4)	−0.012 (3)
O10	0.022 (4)	0.061 (5)	0.066 (5)	−0.005 (3)	0.003 (3)	−0.038 (4)
O11	0.031 (3)	0.027 (4)	0.025 (3)	−0.001 (3)	−0.004 (3)	−0.006 (3)
O12	0.031 (4)	0.079 (6)	0.020 (3)	−0.006 (3)	−0.004 (3)	−0.018 (3)
O13	0.028 (3)	0.033 (4)	0.032 (4)	−0.006 (3)	0.015 (3)	−0.011 (3)
O14	0.034 (4)	0.088 (6)	0.069 (5)	0.000 (4)	−0.009 (4)	0.066 (5)
O15	0.038 (4)	0.046 (4)	0.030 (4)	−0.009 (3)	0.001 (3)	0.017 (3)
O16	0.024 (3)	0.030 (4)	0.034 (4)	0.003 (3)	−0.006 (3)	0.010 (3)
O17	0.024 (3)	0.048 (4)	0.015 (3)	0.007 (3)	0.010 (2)	0.000 (3)
O18	0.021 (3)	0.073 (5)	0.019 (3)	0.016 (3)	−0.012 (3)	−0.012 (3)
O19	0.029 (4)	0.040 (4)	0.029 (4)	0.004 (3)	−0.007 (3)	0.006 (3)
O20	0.033 (4)	0.062 (5)	0.020 (3)	−0.005 (3)	0.004 (3)	0.005 (3)
O21	0.026 (3)	0.023 (3)	0.016 (3)	0.004 (2)	0.001 (2)	0.002 (2)
O22	0.027 (3)	0.013 (3)	0.019 (3)	0.005 (2)	−0.001 (2)	−0.007 (2)
O23	0.023 (3)	0.040 (4)	0.033 (4)	0.005 (3)	0.007 (3)	−0.001 (3)
O24	0.136 (8)	0.038 (5)	0.079 (6)	−0.036 (5)	0.025 (6)	−0.010 (5)
C1	0.030 (5)	0.019 (5)	0.022 (5)	0.009 (4)	0.006 (4)	−0.002 (4)
C2	0.019 (4)	0.017 (4)	0.026 (5)	0.003 (3)	0.004 (3)	0.005 (4)
C3	0.030 (5)	0.033 (6)	0.026 (5)	−0.008 (4)	0.000 (4)	0.004 (4)
C4	0.035 (5)	0.049 (7)	0.026 (5)	−0.009 (5)	−0.001 (4)	−0.001 (5)
C5	0.041 (6)	0.061 (7)	0.020 (5)	−0.024 (5)	0.001 (4)	0.000 (5)
C6	0.027 (5)	0.047 (6)	0.026 (5)	−0.008 (4)	−0.006 (4)	0.003 (4)
C7	0.026 (5)	0.019 (5)	0.017 (4)	0.001 (4)	0.002 (3)	−0.003 (3)
C8	0.033 (5)	0.026 (5)	0.012 (4)	−0.002 (4)	0.000 (3)	0.000 (4)
C9	0.034 (5)	0.048 (6)	0.014 (4)	−0.011 (4)	0.000 (4)	0.001 (4)
C10	0.048 (6)	0.056 (7)	0.027 (5)	−0.020 (5)	−0.007 (5)	0.005 (5)
C11	0.063 (7)	0.038 (6)	0.024 (5)	−0.008 (5)	0.010 (5)	0.005 (5)
C12	0.039 (5)	0.031 (5)	0.018 (5)	−0.011 (4)	0.003 (4)	0.009 (4)
C13	0.022 (4)	0.018 (5)	0.021 (4)	−0.005 (3)	0.005 (4)	−0.002 (3)
C14	0.021 (4)	0.016 (4)	0.025 (5)	−0.006 (3)	0.006 (3)	0.001 (4)
C15	0.036 (5)	0.033 (6)	0.026 (5)	0.008 (4)	−0.003 (4)	−0.005 (4)
C16	0.051 (6)	0.043 (7)	0.025 (5)	0.003 (5)	−0.001 (5)	−0.001 (5)
C17	0.030 (5)	0.030 (6)	0.059 (7)	0.004 (4)	0.020 (5)	0.003 (5)
C18	0.023 (5)	0.029 (5)	0.037 (6)	0.004 (4)	0.005 (4)	0.004 (4)
C19	0.024 (4)	0.021 (5)	0.021 (4)	0.003 (4)	0.001 (3)	−0.004 (4)
C20	0.028 (5)	0.011 (4)	0.026 (5)	0.001 (3)	−0.003 (4)	−0.004 (4)
C21	0.024 (5)	0.020 (5)	0.037 (5)	0.002 (4)	0.003 (4)	0.000 (4)
C22	0.046 (6)	0.022 (5)	0.027 (5)	0.004 (4)	0.009 (4)	0.004 (4)
C23	0.050 (6)	0.028 (5)	0.022 (5)	−0.001 (5)	−0.005 (4)	−0.006 (4)
C24	0.035 (5)	0.031 (6)	0.031 (5)	0.004 (4)	−0.014 (4)	−0.002 (4)
C25	0.017 (4)	0.024 (5)	0.022 (4)	−0.005 (3)	0.000 (3)	−0.003 (4)
C26	0.016 (4)	0.023 (5)	0.035 (5)	0.005 (3)	0.004 (4)	0.005 (4)
C27	0.025 (5)	0.021 (5)	0.014 (4)	0.002 (3)	0.001 (3)	−0.006 (3)
C28	0.025 (4)	0.025 (5)	0.010 (4)	0.002 (4)	0.000 (3)	−0.002 (3)
C29	0.024 (5)	0.044 (6)	0.027 (5)	0.003 (4)	0.000 (4)	−0.001 (4)
C30	0.035 (5)	0.047 (6)	0.017 (5)	0.007 (5)	0.012 (4)	−0.008 (4)
C31	0.050 (7)	0.069 (8)	0.013 (5)	0.004 (6)	−0.018 (4)	−0.010 (5)
C32	0.031 (5)	0.062 (7)	0.021 (5)	0.002 (5)	0.002 (4)	−0.007 (5)
C33	0.030 (5)	0.038 (6)	0.024 (5)	0.011 (4)	−0.002 (4)	0.014 (4)
C34	0.015 (4)	0.027 (5)	0.031 (5)	0.002 (3)	0.003 (4)	0.009 (4)
C35	0.031 (5)	0.033 (6)	0.031 (5)	−0.004 (4)	0.005 (4)	0.004 (4)
C36	0.046 (6)	0.037 (6)	0.029 (5)	−0.015 (5)	−0.007 (4)	0.000 (5)
C37	0.043 (6)	0.025 (5)	0.031 (5)	−0.011 (4)	−0.010 (4)	0.005 (4)
C38	0.031 (5)	0.022 (5)	0.041 (6)	−0.004 (4)	−0.002 (4)	0.006 (4)
C39	0.021 (4)	0.025 (5)	0.017 (4)	−0.005 (4)	0.005 (3)	0.000 (4)
C40	0.015 (4)	0.024 (5)	0.020 (4)	0.000 (3)	−0.003 (3)	0.001 (4)
C41	0.030 (5)	0.028 (5)	0.022 (5)	0.000 (4)	0.002 (4)	0.001 (4)
C42	0.041 (6)	0.041 (6)	0.024 (5)	−0.003 (5)	0.007 (4)	−0.006 (4)
C43	0.035 (5)	0.031 (6)	0.034 (6)	0.004 (4)	−0.008 (4)	−0.001 (4)
C44	0.019 (4)	0.041 (6)	0.029 (5)	−0.006 (4)	−0.003 (4)	−0.005 (4)
C45	0.019 (4)	0.018 (4)	0.023 (5)	−0.007 (3)	−0.002 (4)	0.010 (4)
C46	0.025 (4)	0.018 (4)	0.022 (5)	0.000 (3)	0.000 (3)	0.007 (4)
C47	0.026 (5)	0.034 (5)	0.022 (5)	0.006 (4)	−0.002 (4)	−0.002 (4)
C48	0.031 (5)	0.045 (6)	0.023 (5)	0.013 (4)	−0.007 (4)	0.005 (4)
C49	0.041 (6)	0.036 (6)	0.020 (5)	0.011 (4)	0.010 (4)	0.003 (4)
C50	0.019 (4)	0.031 (5)	0.031 (5)	0.008 (4)	0.001 (4)	0.000 (4)
C51	0.013 (4)	0.026 (5)	0.015 (4)	−0.009 (3)	0.001 (3)	0.008 (4)
C52	0.020 (4)	0.016 (4)	0.018 (4)	−0.002 (3)	−0.001 (3)	−0.003 (3)
C53	0.024 (5)	0.045 (6)	0.029 (5)	0.009 (4)	−0.004 (4)	−0.003 (4)
C54	0.045 (6)	0.051 (7)	0.025 (5)	0.006 (5)	0.006 (5)	−0.017 (5)
C55	0.043 (6)	0.034 (6)	0.026 (5)	−0.007 (5)	−0.007 (4)	−0.007 (4)
C56	0.030 (5)	0.038 (6)	0.020 (5)	0.005 (4)	−0.003 (4)	−0.004 (4)
N1	0.035 (4)	0.043 (5)	0.022 (4)	−0.013 (4)	−0.001 (3)	0.000 (4)
N2	0.051 (5)	0.049 (5)	0.018 (4)	−0.016 (4)	−0.001 (4)	−0.005 (4)
N3	0.055 (6)	0.044 (6)	0.033 (5)	−0.012 (4)	0.020 (4)	−0.006 (4)
N4	0.057 (5)	0.038 (5)	0.012 (4)	0.007 (4)	0.001 (4)	−0.007 (3)
N5	0.028 (4)	0.036 (5)	0.030 (4)	−0.014 (3)	−0.002 (3)	0.006 (4)
N6	0.048 (5)	0.036 (5)	0.019 (4)	−0.005 (4)	−0.004 (4)	0.001 (3)
N7	0.042 (5)	0.045 (5)	0.017 (4)	0.011 (4)	−0.004 (3)	−0.002 (4)
N8	0.048 (5)	0.035 (5)	0.018 (4)	−0.007 (4)	0.002 (4)	−0.008 (4)

### Geometric parameters (Å, °)

**Table:**

La1—O6	2.420 (6)	C4—N1	1.342 (11)
La1—O4^i^	2.434 (5)	C4—H4A	0.9300
La1—O3	2.445 (5)	C5—N1	1.334 (11)
La1—O8	2.451 (6)	C5—C6	1.370 (12)
La1—O5^ii^	2.486 (6)	C5—H5A	0.9300
La1—O1	2.494 (5)	C6—H6A	0.9300
La1—O2^ii^	2.545 (5)	C7—C8	1.480 (11)
La1—O7	2.703 (6)	C8—C12	1.374 (11)
La2—O9	2.399 (6)	C8—C9	1.383 (11)
La2—O17	2.404 (5)	C9—C10	1.373 (12)
La2—O13	2.443 (6)	C9—H9A	0.9300
La2—O19	2.444 (6)	C10—N2	1.336 (12)
La2—O11	2.462 (5)	C10—H10A	0.9300
La2—O15	2.515 (6)	C11—N2	1.326 (12)
La2—O16	2.606 (5)	C11—C12	1.369 (12)
La2—O22^iii^	2.607 (5)	C11—H11A	0.9300
La3—O18	2.387 (5)	C12—H12A	0.9300
La3—O14^iv^	2.436 (6)	C13—C14	1.505 (11)
La3—O20	2.436 (6)	C14—C15	1.369 (11)
La3—O12^iv^	2.439 (6)	C14—C18	1.378 (11)
La3—O10^iv^	2.515 (6)	C15—C16	1.371 (12)
La3—O21	2.574 (5)	C15—H15A	0.9300
La3—O24	2.610 (7)	C16—N3	1.319 (12)
La3—O23	2.657 (6)	C16—H16A	0.9300
La3—O11^iv^	2.797 (6)	C17—N3	1.338 (13)
La3—C26^iv^	3.043 (8)	C17—C18	1.377 (13)
Cu1—N4^v^	1.920 (7)	C17—H17A	0.9300
Cu1—N2	1.931 (7)	C18—H18A	0.9300
Cu2—Cu2'	0.666 (13)	C19—C20	1.516 (11)
Cu2—N3	1.917 (9)	C20—C25	1.390 (11)
Cu2—Br1	2.212 (4)	C20—C21	1.405 (11)
Cu2—Cu7^iv^	2.770 (4)	C21—C22	1.384 (12)
Cu2'—N3	2.064 (18)	C21—H21A	0.9300
Cu2'—Br1	2.415 (16)	C22—C23	1.357 (12)
Cu2'—Cu7^iv^	2.569 (17)	C22—H22A	0.9300
Cu2'—Br3^iv^	2.572 (16)	C23—C24	1.393 (12)
Cu3—Cu3'	0.629 (14)	C23—H23A	0.9300
Cu3—N1^vi^	1.959 (8)	C24—C25	1.400 (11)
Cu3—N7^vii^	1.980 (8)	C24—H24A	0.9300
Cu3—Br2	2.609 (4)	C25—C26	1.476 (11)
Cu3'—N7^vii^	1.927 (19)	C26—La3^vii^	3.043 (8)
Cu3'—N1^vi^	1.976 (19)	C27—C28	1.493 (10)
Cu3'—Br3	2.72 (2)	C28—C29	1.380 (11)
Cu4—Br3	2.3071 (19)	C28—C32	1.399 (11)
Cu4—Br2	2.328 (2)	C29—C30	1.376 (11)
Cu4—Br4	2.3533 (19)	C29—H29A	0.9300
Cu4—Cu5	2.761 (2)	C30—N4	1.311 (11)
Cu5—N5	2.005 (7)	C30—H30A	0.9300
Cu5—Br5	2.3441 (16)	C31—N4	1.347 (12)
Cu5—Br4	2.3827 (16)	C31—C32	1.369 (12)
Cu6—N8^viii^	2.004 (7)	C31—H31A	0.9300
Cu6—Br5	2.4397 (17)	C32—H32A	0.9300
Cu6—Br3	2.4946 (18)	C33—C34	1.490 (11)
Cu6—Br6	2.5481 (17)	C34—C38	1.364 (11)
Cu7—N6^viii^	2.037 (7)	C34—C35	1.381 (12)
Cu7—Br6	2.4415 (18)	C35—C36	1.389 (12)
Cu7—Br1^vii^	2.4447 (18)	C35—H35A	0.9300
Cu7—Cu2'^vii^	2.569 (17)	C36—N5	1.335 (11)
Cu7—Br4^vii^	2.6722 (19)	C36—H36A	0.9300
Cu7—Cu2^vii^	2.770 (4)	C37—N5	1.326 (11)
Br1—Cu7^iv^	2.4447 (18)	C37—C38	1.386 (12)
Br3—Cu2'^vii^	2.572 (17)	C37—H37A	0.9300
Br4—Cu7^iv^	2.6722 (19)	C38—H38A	0.9300
O1—C1	1.253 (9)	C39—C40	1.491 (11)
O2—C1	1.240 (9)	C40—C44	1.380 (11)
O2—La1^ii^	2.545 (5)	C40—C41	1.382 (11)
O3—C7	1.268 (9)	C41—C42	1.389 (12)
O4—C7	1.247 (9)	C41—H41A	0.9300
O4—La1^i^	2.434 (5)	C42—N6	1.342 (12)
O5—C13	1.247 (9)	C42—H42A	0.9300
O5—La1^ii^	2.486 (6)	C43—N6	1.332 (12)
O6—C13	1.236 (9)	C43—C44	1.354 (12)
O7—H7A	0.8505	C43—H43A	0.9300
O7—H7B	0.8512	C44—H44A	0.9300
O8—C19	1.232 (10)	C45—C46	1.507 (11)
O9—C19	1.244 (10)	C46—C47	1.383 (11)
O10—C26	1.247 (9)	C46—C50	1.388 (11)
O10—La3^vii^	2.515 (6)	C47—C48	1.374 (11)
O11—C26	1.279 (10)	C47—H47A	0.9300
O11—La3^vii^	2.797 (6)	C48—N7	1.324 (11)
O12—C27	1.243 (9)	C48—H48A	0.9300
O12—La3^vii^	2.439 (6)	C49—N7	1.317 (11)
O13—C27	1.256 (9)	C49—C50	1.377 (12)
O14—C33	1.250 (10)	C49—H49A	0.9300
O14—La3^vii^	2.436 (6)	C50—H50A	0.9300
O15—C33	1.237 (10)	C51—C52	1.493 (10)
O16—H16C	0.8495	C52—C53	1.384 (11)
O16—H16D	0.8589	C52—C56	1.393 (11)
O17—C39	1.243 (9)	C53—C54	1.371 (12)
O18—C39	1.250 (9)	C53—H53A	0.9300
O19—C45	1.235 (9)	C54—N8	1.343 (12)
O20—C45	1.241 (10)	C54—H54A	0.9300
O21—C51	1.258 (9)	C55—N8	1.328 (11)
O22—C51	1.252 (9)	C55—C56	1.379 (12)
O22—La2^iii^	2.607 (5)	C55—H55A	0.9300
O23—H23C	0.8013	C56—H56A	0.9300
O23—H23D	0.9328	N1—Cu3^v^	1.959 (8)
O24—H24C	0.8501	N1—Cu3'^v^	1.976 (19)
O24—H24D	0.8498	N4—Cu1^vi^	1.920 (7)
C1—C2	1.521 (11)	N6—Cu7^ix^	2.037 (7)
C2—C6	1.375 (11)	N7—Cu3'^iv^	1.927 (19)
C2—C3	1.380 (11)	N7—Cu3^iv^	1.980 (8)
C3—C4	1.390 (12)	N8—Cu6^ix^	2.004 (7)
C3—H3A	0.9300		
			
O6—La1—O4^i^	150.8 (2)	H23C—O23—H23D	103.4
O6—La1—O3	92.4 (2)	La3—O24—H24C	162.5
O4^i^—La1—O3	85.15 (19)	La3—O24—H24D	89.5
O6—La1—O8	75.4 (2)	H24C—O24—H24D	107.7
O4^i^—La1—O8	75.8 (2)	O2—C1—O1	127.2 (8)
O3—La1—O8	74.6 (2)	O2—C1—C2	117.0 (7)
O6—La1—O5^ii^	115.8 (2)	O1—C1—C2	115.7 (7)
O4^i^—La1—O5^ii^	82.4 (2)	C6—C2—C3	115.9 (8)
O3—La1—O5^ii^	140.6 (2)	C6—C2—C1	123.3 (8)
O8—La1—O5^ii^	136.6 (2)	C3—C2—C1	120.8 (7)
O6—La1—O1	72.8 (2)	C2—C3—C4	120.1 (8)
O4^i^—La1—O1	134.92 (19)	C2—C3—H3A	119.9
O3—La1—O1	80.7 (2)	C4—C3—H3A	119.9
O8—La1—O1	138.4 (2)	N1—C4—C3	122.8 (8)
O5^ii^—La1—O1	82.1 (2)	N1—C4—H4A	118.6
O6—La1—O2^ii^	75.5 (2)	C3—C4—H4A	118.6
O4^i^—La1—O2^ii^	91.89 (18)	N1—C5—C6	122.6 (9)
O3—La1—O2^ii^	149.2 (2)	N1—C5—H5A	118.7
O8—La1—O2^ii^	74.9 (2)	C6—C5—H5A	118.7
O5^ii^—La1—O2^ii^	68.7 (2)	C5—C6—C2	121.7 (8)
O1—La1—O2^ii^	120.78 (18)	C5—C6—H6A	119.1
O6—La1—O7	138.0 (2)	C2—C6—H6A	119.1
O4^i^—La1—O7	68.51 (19)	O4—C7—O3	125.2 (7)
O3—La1—O7	71.97 (19)	O4—C7—C8	117.0 (7)
O8—La1—O7	132.38 (19)	O3—C7—C8	117.7 (7)
O5^ii^—La1—O7	68.7 (2)	C12—C8—C9	117.5 (8)
O1—La1—O7	66.41 (19)	C12—C8—C7	120.8 (8)
O2^ii^—La1—O7	134.9 (2)	C9—C8—C7	121.7 (8)
O9—La2—O17	76.8 (2)	C10—C9—C8	119.3 (8)
O9—La2—O13	147.7 (2)	C10—C9—H9A	120.3
O17—La2—O13	135.3 (2)	C8—C9—H9A	120.3
O9—La2—O19	88.4 (2)	N2—C10—C9	123.3 (9)
O17—La2—O19	87.0 (2)	N2—C10—H10A	118.3
O13—La2—O19	89.7 (2)	C9—C10—H10A	118.3
O9—La2—O11	72.6 (2)	N2—C11—C12	124.1 (9)
O17—La2—O11	109.47 (19)	N2—C11—H11A	117.9
O13—La2—O11	94.55 (19)	C12—C11—H11A	117.9
O19—La2—O11	150.5 (2)	C11—C12—C8	119.2 (9)
O9—La2—O15	77.8 (2)	C11—C12—H12A	120.4
O17—La2—O15	150.7 (2)	C8—C12—H12A	120.4
O13—La2—O15	70.3 (2)	O6—C13—O5	124.6 (8)
O19—La2—O15	77.8 (2)	O6—C13—C14	118.9 (7)
O11—La2—O15	76.2 (2)	O5—C13—C14	116.6 (7)
O9—La2—O16	118.8 (2)	C15—C14—C18	118.7 (8)
O17—La2—O16	77.99 (19)	C15—C14—C13	119.3 (8)
O13—La2—O16	79.00 (19)	C18—C14—C13	122.0 (8)
O19—La2—O16	144.06 (19)	C14—C15—C16	119.3 (9)
O11—La2—O16	65.12 (18)	C14—C15—H15A	120.3
O15—La2—O16	127.83 (19)	C16—C15—H15A	120.3
O9—La2—O22^iii^	140.9 (2)	N3—C16—C15	122.9 (9)
O17—La2—O22^iii^	66.62 (19)	N3—C16—H16A	118.5
O13—La2—O22^iii^	69.22 (17)	C15—C16—H16A	118.5
O19—La2—O22^iii^	76.68 (18)	N3—C17—C18	123.1 (9)
O11—La2—O22^iii^	131.93 (18)	N3—C17—H17A	118.5
O15—La2—O22^iii^	131.72 (19)	C18—C17—H17A	118.5
O16—La2—O22^iii^	67.40 (17)	C17—C18—C14	118.2 (9)
O18—La3—O14^iv^	144.2 (3)	C17—C18—H18A	120.9
O18—La3—O20	89.3 (2)	C14—C18—H18A	120.9
O14^iv^—La3—O20	83.5 (2)	O8—C19—O9	124.5 (8)
O18—La3—O12^iv^	139.1 (2)	O8—C19—C20	117.4 (7)
O14^iv^—La3—O12^iv^	76.4 (3)	O9—C19—C20	118.1 (8)
O20—La3—O12^iv^	91.9 (2)	C25—C20—C21	119.1 (8)
O18—La3—O10^iv^	75.0 (2)	C25—C20—C19	124.9 (7)
O14^iv^—La3—O10^iv^	85.1 (3)	C21—C20—C19	115.8 (7)
O20—La3—O10^iv^	134.2 (2)	C22—C21—C20	120.9 (8)
O12^iv^—La3—O10^iv^	127.9 (2)	C22—C21—H21A	119.5
O18—La3—O21	75.0 (2)	C20—C21—H21A	119.5
O14^iv^—La3—O21	131.0 (2)	C23—C22—C21	120.1 (9)
O20—La3—O21	137.6 (2)	C23—C22—H22A	120.0
O12^iv^—La3—O21	76.8 (2)	C21—C22—H22A	120.0
O10^iv^—La3—O21	80.0 (2)	C22—C23—C24	120.1 (9)
O18—La3—O24	70.9 (3)	C22—C23—H23A	120.0
O14^iv^—La3—O24	73.9 (3)	C24—C23—H23A	120.0
O20—La3—O24	68.5 (3)	C23—C24—C25	120.9 (8)
O12^iv^—La3—O24	145.8 (3)	C23—C24—H24A	119.5
O10^iv^—La3—O24	65.7 (3)	C25—C24—H24A	119.5
O21—La3—O24	136.4 (2)	C20—C25—C24	118.8 (8)
O18—La3—O23	74.5 (2)	C20—C25—C26	124.2 (7)
O14^iv^—La3—O23	132.5 (3)	C24—C25—C26	117.0 (7)
O20—La3—O23	67.4 (2)	O10—C26—O11	120.5 (8)
O12^iv^—La3—O23	68.4 (2)	O10—C26—C25	118.7 (8)
O10^iv^—La3—O23	141.9 (2)	O11—C26—C25	120.6 (7)
O21—La3—O23	70.47 (17)	O10—C26—La3^vii^	53.7 (4)
O24—La3—O23	123.1 (3)	O11—C26—La3^vii^	66.8 (4)
O18—La3—O11^iv^	115.82 (19)	C25—C26—La3^vii^	171.5 (6)
O14^iv^—La3—O11^iv^	66.7 (2)	O12—C27—O13	124.9 (7)
O20—La3—O11^iv^	150.15 (19)	O12—C27—C28	117.7 (7)
O12^iv^—La3—O11^iv^	79.61 (18)	O13—C27—C28	117.4 (7)
O10^iv^—La3—O11^iv^	48.42 (18)	C29—C28—C32	116.4 (8)
O21—La3—O11^iv^	68.52 (17)	C29—C28—C27	122.2 (7)
O24—La3—O11^iv^	103.3 (2)	C32—C28—C27	121.4 (7)
O23—La3—O11^iv^	132.43 (17)	C30—C29—C28	119.9 (8)
O18—La3—C26^iv^	95.1 (2)	C30—C29—H29A	120.1
O14^iv^—La3—C26^iv^	74.9 (3)	C28—C29—H29A	120.1
O20—La3—C26^iv^	148.7 (2)	N4—C30—C29	124.0 (8)
O12^iv^—La3—C26^iv^	104.4 (2)	N4—C30—H30A	118.0
O10^iv^—La3—C26^iv^	23.6 (2)	C29—C30—H30A	118.0
O21—La3—C26^iv^	73.01 (19)	N4—C31—C32	123.2 (8)
O24—La3—C26^iv^	83.7 (3)	N4—C31—H31A	118.4
O23—La3—C26^iv^	143.5 (2)	C32—C31—H31A	118.4
O11^iv^—La3—C26^iv^	24.84 (19)	C31—C32—C28	119.6 (9)
N4^v^—Cu1—N2	166.2 (3)	C31—C32—H32A	120.2
Cu2'—Cu2—N3	93.2 (18)	C28—C32—H32A	120.2
Cu2'—Cu2—Br1	99.8 (18)	O15—C33—O14	126.0 (8)
N3—Cu2—Br1	166.5 (3)	O15—C33—C34	119.1 (7)
Cu2'—Cu2—Cu7^iv^	65.7 (18)	O14—C33—C34	114.9 (8)
N3—Cu2—Cu7^iv^	133.0 (3)	C38—C34—C35	118.3 (8)
Br1—Cu2—Cu7^iv^	57.48 (10)	C38—C34—C33	121.0 (8)
Cu2—Cu2'—N3	68.0 (16)	C35—C34—C33	120.6 (8)
Cu2—Cu2'—Br1	64.5 (16)	C34—C35—C36	119.0 (8)
N3—Cu2'—Br1	132.4 (7)	C34—C35—H35A	120.5
Cu2—Cu2'—Cu7^iv^	101 (2)	C36—C35—H35A	120.5
N3—Cu2'—Cu7^iv^	136.8 (10)	N5—C36—C35	123.1 (9)
Br1—Cu2'—Cu7^iv^	58.6 (4)	N5—C36—H36A	118.5
Cu2—Cu2'—Br3^iv^	152 (3)	C35—C36—H36A	118.5
N3—Cu2'—Br3^iv^	113.9 (7)	N5—C37—C38	124.2 (8)
Br1—Cu2'—Br3^iv^	106.3 (7)	N5—C37—H37A	117.9
Cu7^iv^—Cu2'—Br3^iv^	95.3 (5)	C38—C37—H37A	117.9
Cu3'—Cu3—N1^vi^	82.3 (19)	C34—C38—C37	118.7 (9)
Cu3'—Cu3—N7^vii^	76.0 (19)	C34—C38—H38A	120.6
N1^vi^—Cu3—N7^vii^	148.7 (4)	C37—C38—H38A	120.6
Cu3'—Cu3—Br2	147 (2)	O17—C39—O18	123.8 (7)
N1^vi^—Cu3—Br2	106.5 (3)	O17—C39—C40	118.7 (7)
N7^vii^—Cu3—Br2	104.0 (3)	O18—C39—C40	117.5 (7)
Cu3—Cu3'—N7^vii^	86 (2)	C44—C40—C41	119.4 (8)
Cu3—Cu3'—N1^vi^	79.3 (19)	C44—C40—C39	120.2 (7)
N7^vii^—Cu3'—N1^vi^	152.7 (12)	C41—C40—C39	120.4 (7)
Cu3—Cu3'—Br3	109 (2)	C40—C41—C42	118.7 (8)
N7^vii^—Cu3'—Br3	92.9 (7)	C40—C41—H41A	120.7
N1^vi^—Cu3'—Br3	113.5 (9)	C42—C41—H41A	120.7
Br3—Cu4—Br2	114.05 (8)	N6—C42—C41	121.9 (9)
Br3—Cu4—Br4	125.56 (8)	N6—C42—H42A	119.0
Br2—Cu4—Br4	120.39 (8)	C41—C42—H42A	119.0
Br3—Cu4—Cu5	82.39 (6)	N6—C43—C44	125.1 (9)
Br2—Cu4—Cu5	142.32 (8)	N6—C43—H43A	117.5
Br4—Cu4—Cu5	54.84 (5)	C44—C43—H43A	117.5
N5—Cu5—Br5	120.8 (2)	C43—C44—C40	117.7 (9)
N5—Cu5—Br4	108.5 (2)	C43—C44—H44A	121.2
Br5—Cu5—Br4	130.48 (6)	C40—C44—H44A	121.2
N5—Cu5—Cu4	88.5 (2)	O19—C45—O20	125.8 (8)
Br5—Cu5—Cu4	127.83 (7)	O19—C45—C46	117.5 (8)
Br4—Cu5—Cu4	53.85 (5)	O20—C45—C46	116.7 (7)
N8^viii^—Cu6—Br5	119.7 (2)	C47—C46—C50	117.3 (8)
N8^viii^—Cu6—Br3	106.4 (2)	C47—C46—C45	121.1 (8)
Br5—Cu6—Br3	109.80 (6)	C50—C46—C45	121.5 (7)
N8^viii^—Cu6—Br6	113.8 (2)	C48—C47—C46	118.9 (8)
Br5—Cu6—Br6	105.98 (6)	C48—C47—H47A	120.6
Br3—Cu6—Br6	99.15 (6)	C46—C47—H47A	120.6
N6^viii^—Cu7—Br6	109.2 (2)	N7—C48—C47	124.0 (8)
N6^viii^—Cu7—Br1^vii^	108.6 (2)	N7—C48—H48A	118.0
Br6—Cu7—Br1^vii^	129.77 (7)	C47—C48—H48A	118.0
N6^viii^—Cu7—Cu2'^vii^	130.7 (5)	N7—C49—C50	124.0 (8)
Br6—Cu7—Cu2'^vii^	72.8 (4)	N7—C49—H49A	118.0
Br1^vii^—Cu7—Cu2'^vii^	57.5 (4)	C50—C49—H49A	118.0
N6^viii^—Cu7—Br4^vii^	98.8 (2)	C49—C50—C46	118.9 (8)
Br6—Cu7—Br4^vii^	102.53 (6)	C49—C50—H50A	120.5
Br1^vii^—Cu7—Br4^vii^	103.04 (6)	C46—C50—H50A	120.5
Cu2'^vii^—Cu7—Br4^vii^	129.6 (4)	O22—C51—O21	125.4 (7)
N6^viii^—Cu7—Cu2^vii^	140.1 (3)	O22—C51—C52	118.1 (7)
Br6—Cu7—Cu2^vii^	80.14 (9)	O21—C51—C52	116.6 (7)
Br1^vii^—Cu7—Cu2^vii^	49.72 (9)	C53—C52—C56	116.2 (7)
Cu2'^vii^—Cu7—Cu2^vii^	13.7 (3)	C53—C52—C51	123.1 (7)
Br4^vii^—Cu7—Cu2^vii^	117.39 (9)	C56—C52—C51	120.6 (7)
Cu2—Br1—Cu2'	15.8 (3)	C54—C53—C52	121.2 (8)
Cu2—Br1—Cu7^iv^	72.81 (11)	C54—C53—H53A	119.4
Cu2'—Br1—Cu7^iv^	63.8 (4)	C52—C53—H53A	119.4
Cu4—Br2—Cu3	82.10 (12)	N8—C54—C53	121.9 (9)
Cu4—Br3—Cu6	104.81 (7)	N8—C54—H54A	119.0
Cu4—Br3—Cu2'^vii^	158.6 (4)	C53—C54—H54A	119.0
Cu6—Br3—Cu2'^vii^	91.1 (4)	N8—C55—C56	123.3 (9)
Cu4—Br3—Cu3'	85.6 (3)	N8—C55—H55A	118.3
Cu6—Br3—Cu3'	156.9 (4)	C56—C55—H55A	118.3
Cu2'^vii^—Br3—Cu3'	85.1 (5)	C55—C56—C52	119.6 (8)
Cu4—Br4—Cu5	71.31 (6)	C55—C56—H56A	120.2
Cu4—Br4—Cu7^iv^	120.66 (6)	C52—C56—H56A	120.2
Cu5—Br4—Cu7^iv^	123.90 (6)	C5—N1—C4	116.9 (8)
Cu5—Br5—Cu6	84.24 (6)	C5—N1—Cu3^v^	121.7 (6)
Cu7—Br6—Cu6	98.64 (6)	C4—N1—Cu3^v^	121.3 (6)
C1—O1—La1	136.1 (6)	C5—N1—Cu3'^v^	132.7 (8)
C1—O2—La1^ii^	143.8 (5)	C4—N1—Cu3'^v^	109.2 (8)
C7—O3—La1	145.1 (5)	Cu3^v^—N1—Cu3'^v^	18.4 (4)
C7—O4—La1^i^	152.6 (6)	C11—N2—C10	116.4 (8)
C13—O5—La1^ii^	116.1 (5)	C11—N2—Cu1	119.1 (6)
C13—O6—La1	165.8 (6)	C10—N2—Cu1	124.4 (6)
La1—O7—H7A	99.1	C16—N3—C17	117.6 (8)
La1—O7—H7B	102.7	C16—N3—Cu2	122.9 (7)
H7A—O7—H7B	107.5	C17—N3—Cu2	119.4 (7)
C19—O8—La1	163.1 (6)	C16—N3—Cu2'	104.2 (8)
C19—O9—La2	146.1 (6)	C17—N3—Cu2'	137.9 (8)
C26—O10—La3^vii^	102.7 (5)	Cu2—N3—Cu2'	18.8 (4)
C26—O11—La2	146.8 (5)	C30—N4—C31	116.8 (7)
C26—O11—La3^vii^	88.4 (5)	C30—N4—Cu1^vi^	123.0 (6)
La2—O11—La3^vii^	123.8 (2)	C31—N4—Cu1^vi^	120.2 (6)
C27—O12—La3^vii^	150.7 (6)	C37—N5—C36	116.5 (8)
C27—O13—La2	129.7 (5)	C37—N5—Cu5	119.6 (6)
C33—O14—La3^vii^	143.8 (7)	C36—N5—Cu5	123.6 (6)
C33—O15—La2	141.0 (6)	C43—N6—C42	117.2 (8)
La2—O16—H16C	150.9	C43—N6—Cu7^ix^	121.8 (6)
La2—O16—H16D	101.5	C42—N6—Cu7^ix^	121.0 (6)
H16C—O16—H16D	107.0	C49—N7—C48	116.8 (7)
C39—O17—La2	151.1 (6)	C49—N7—Cu3'^iv^	111.3 (8)
C39—O18—La3	147.0 (5)	C48—N7—Cu3'^iv^	127.6 (9)
C45—O19—La2	150.1 (6)	C49—N7—Cu3^iv^	123.6 (6)
C45—O20—La3	154.4 (6)	C48—N7—Cu3^iv^	119.4 (6)
C51—O21—La3	129.1 (5)	Cu3'^iv^—N7—Cu3^iv^	18.5 (4)
C51—O22—La2^iii^	126.3 (5)	C55—N8—C54	117.7 (8)
La3—O23—H23C	114.8	C55—N8—Cu6^ix^	121.1 (6)
La3—O23—H23D	115.0	C54—N8—Cu6^ix^	121.0 (6)

Symmetry codes: (i) −*x*+1, −*y*+1, −*z*; (ii) −*x*, −*y*+1, −*z*; (iii) −*x*, −*y*, −*z*; (iv) *x*−1, *y*, *z*; (v) −*x*+1, *y*+1/2, −*z*−1/2; (vi) −*x*+1, *y*−1/2, −*z*−1/2; (vii) *x*+1, *y*, *z*; (viii) *x*+1, −*y*+1/2, *z*−1/2; (ix) *x*−1, −*y*+1/2, *z*+1/2.

### Hydrogen-bond geometry (Å, °)

**Table:**

*D*—H···*A*	*D*—H	H···*A*	*D*···*A*	*D*—H···*A*
O16—H16D···O21^vii^	0.86	2.09	2.901 (7)	157
O23—H23D···O22	0.93	2.00	2.861 (8)	153
O24—H24D···O20	0.85	2.22	2.844 (11)	130

Symmetry codes: (vii) *x*+1, *y*, *z*.
